# A millisecond micro-RNA separation technique by a hybrid structure of nanopillars and nanoslits

**DOI:** 10.1038/srep43877

**Published:** 2017-03-08

**Authors:** Qiong Wu, Noritada Kaji, Takao Yasui, Sakon Rahong, Takeshi Yanagida, Masaki Kanai, Kazuki Nagashima, Manabu Tokeshi, Tomoji Kawai, Yoshinobu Baba

**Affiliations:** 1 Department of Applied Chemistry, Graduate School of Engineering, Nagoya University, Nagoya 464-8603, Japan; 2 ImPACT Research Center for Advanced Nanobiodevices, Nagoya University, Nagoya 464-8603, Japan; 3 JST, PRESTO, Furo-cho, Chikusa-ku, Nagoya 464-8603, Japan; 4 Institute for Materials Chemistry and Engineering, Kyushu University, Fukuoka 816-8580, Japan; 5 Division of Biotechnology and Macromolecular Chemistry, Faculty of Engineering, Hokkaido University, Kita 13 Nishi 8, Kita-ku, Sapporo 060-8628, Japan; 6 Institute of Scientific and Industrial Research, Osaka University, 8-1Mihogaoka-cho, Ibaraki, Osaka 567-0047, Japan; 7 Health Research Institute, National Institute of Advanced Industrial Science and Technology (AIST), Takamatsu 761-0395, Japan; 8 College of Pharmacy, Kaohsiung Medical University 100, Shih-Chuan 1st Rd., Kaohsiung, 807, Taiwan, R.O.C

## Abstract

A millisecond micro-RNA separation of a mixture of total RNA and genomic DNA, extracted from cultured HeLa cells, was successfully achieved using a hybrid structure of nanopillars and nanoslits contained inside a microchannel. The nanopillars, 250-nm in diameter and 100-nm in height, were fabricated with a 750-nm space inside the nanoslits, which were 100-nm in height and 25-μm in width; the nanopillars were then applied as a new sieve matrix. This ultra-fast technique for the separation of miRNA can be an effective pretreatment for semiconductor nanopore DNA sequencing, which has an optimum reading speed of 1 base/ms to obtain effective signal-to-noise ratio and discriminate each base by ion or tunneling current during the passage of nucleic acids.

MiRNAs are approximately 22-nucleotide-long, non-coding, single-stranded RNA molecules that are processed from a longer double-stranded precursor miRNA (pre-miRNA) by a ribonuclease III known as Dicer. In contrast with messenger RNA, the short length of miRNAs endows them with excellent stability, resistance to mechanical and enzymatic degradation, and ability to circulate in bodily fluids for an extended time^1^. The levels of miRNA in bodily fluids, including blood, saliva, and urine, are strongly correlated with initiation and progression of cancer^2^,^3^. For this reason, miRNAs can be used as easy-to-acquire, nearly non-invasive potential biomarkers for the diagnosis and prognosis of cancer. The commercially available reagents for the extraction of miRNA include PureLink^TM^ miRNA Isolation Kit (Invitrogen, Life Technologies), mirVana^TM^ miRNA Isolation Kit (Ambion, Life Technologies), and miRNeasy Serum/Plasma Kit (Qiagen). The principles of miRNA extraction are similar; samples are disrupted in a denaturing lysis buffer, followed by an acid-phenol chloroform extraction that removes most of the cellular components except the RNA molecules. After this liquid-liquid extraction, all the species of RNA present in the aqueous phase, including miRNA, rRNA, tRNA, and mRNA, are bound on a silica-based membrane. Selective binding and purification of small RNA molecules on the membrane is achieved by using an optimum ethanol concentration^4^,^5^. Although these kits are optimized to minimize the manual-handling processes and the quantity of reagents, the offline processes can be labor intensive. The process of extracting miRNA is crucial for the consequent steps of quantification and single-chip sequencing, and ultimately determines the quality of the obtained data.

Microchip electrophoresis has been greatly improved with respect to speed of separation, resolution, and applicability, such as that for detection of miRNA; however, several issues need to be resolved, such as minimizing injection-band broadening and length of separation, and maximizing the applied electric field. Jacobson *et al*. used a microchannel 26-μm in width and 7.0-μm in depth to generate a narrow injection band^6^, achieving a successful separation of a mixture of rhodamine B and dichlorofluorescein in 0.8 ms using an electric field of 53 kV cm^−1^ and a separation length of 200 μm. Short separation time of less than 1.0 ms resulted in negligible contribution from thermal diffusion by Joule heating at a high electric field. The narrow capillary of approximately 26-μm i.d. allowed for the use of greater field strengths; 120 kV cm^−1^ and 20-μs separation of 5-hydroxyindoles and 5-hydroxytryptophan was achieved using a separation length of 10-μm by combined optical gating injection method^7^,^8^. However, these targets for separation have all been substances with a low molecular weight rather than nucleic acids or proteins. Using capillary and microchip electrophoresis has allowed seconds-long separation of nucleic acids and protein molecules; however, there is still the unrealized goal of more efficient separation that would require only milli or submilliseconds.

The nanopore-based DNA sequencer is a promising next-generation DNA and RNA sequencing technology, which will enable reading base sequences directly from a single DNA or RNA molecule, and will eliminate amplification and synthesis of cDNA. Nanopore-based DNA sequencing has an optimum reading speed of 1 base/ms to obtain an effective signal-to-noise ratio of an ion or tunneling current for discrimination of each base during the passage of nucleic acids^9^,^10^. To achieve the sequencing of whole DNA or RNA based on the reading speed of 1 base/ms, each step in this process, including the extraction of nucleic acids from cells, separation of nucleic acids based on size, and introduction into nanopores, should be done at the level of ms. Because the entire process of miRNA analysis is conducted in a semiconductor nanopore-based sequencer, we developed a technique for rapid miRNA separation from a mixture of various sizes of nucleic acids using a hybrid structure of nanopillars^11^,^12^,^13^ and nanoslits^14^,^15^,^16^.

Nanopillars, which have nanometer-scale pillar structures, were fabricated inside microchannels using various spacing, and were then used as matrices for DNA and protein separation instead of natural or synthetic polymers^13^,^17^. This separation approach using top-down fabrication technique enabled the precise control of DNA conformation during electrophoresis and demonstrated that the geometric pattern of the nanopillar array can control the separation mode and enhance the throughput^18^,^19^. The square pattern improved the resolution of separation proportionally to the applied electric field and transferred the larger DNA molecules more rapidly than it transferred the smaller ones. Combining nanoslit structures can provide an entropic trapping effect^15^,^16^ and improve the speed of separation and resolution. Feasibility studies, using a mixture of total RNA and genomic DNA, were performed to elucidate whether this technique is applicable with a wide size range of nucleic acids. As a result, a mixture of genomic DNA, total RNA, and miRNA from HeLa cells was separated only within 100 ms.

## Results and Discussion

### Design of the device

**Figure 1:**
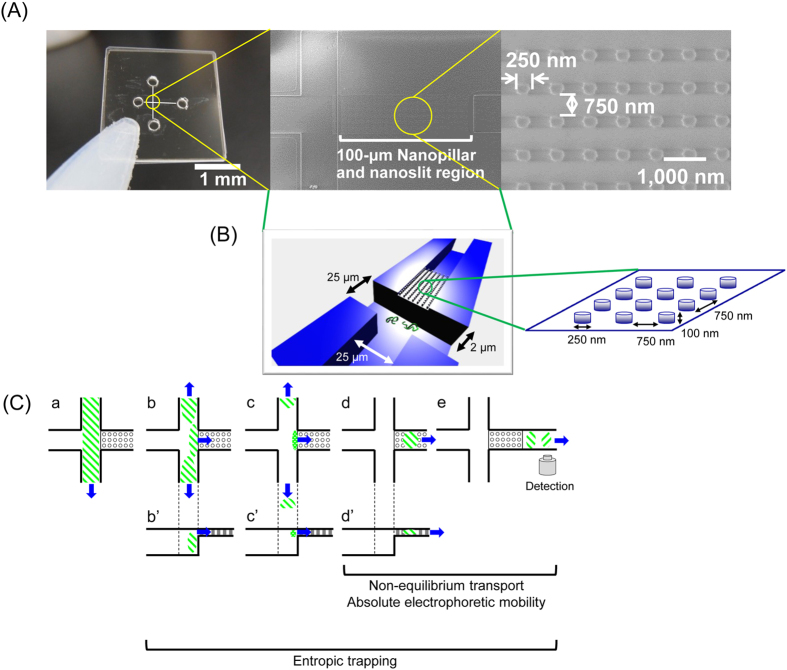
(**A**) An image of the nanobiodevice (left), an SEM image of the hybrid structure of nanopillars and nanoslits (middle), and the magnified SEM image (right). (**B**) Schematic of the hybrid structure of nanopillars and nanoslits around the cross injector. (**C**) Diagram showing the scheme of sample injection. Figure panels C-a to C-e are arranged by time in ascending order. Sample of nucleic acids and direction of electrophoretic migration are depicted in green diagonal and blue arrows, respectively. Suggested dominant mechanisms for each process are shown at the bottom.

As shown in Fig. 1, nanopillars, 250 nm in diameter and 100 nm in height, were fabricated with 750-nm spacing inside a nanoslit structure, 100 nm in height and 100 μm in length. This structure was fabricated inside a 25-μm wide microchannel without any offset from the cross injector; hence, separation could begin without lag after switching the electric field from the sample-loading mode to separation mode that was based on the pinched injection scheme. Because the structures were fabricated using quartz, high tolerance for a strong electric field was an advantage for performing high-speed separation. We hypothesized that non-equilibrium transport using nanopillars with a square array pattern, and entropic trapping under a high electric field by the nanoslits, would accelerate separation and enhance the resolution of separation.

### Separation of mixtures containing DNA markers and miRNA

**Figure 2:**
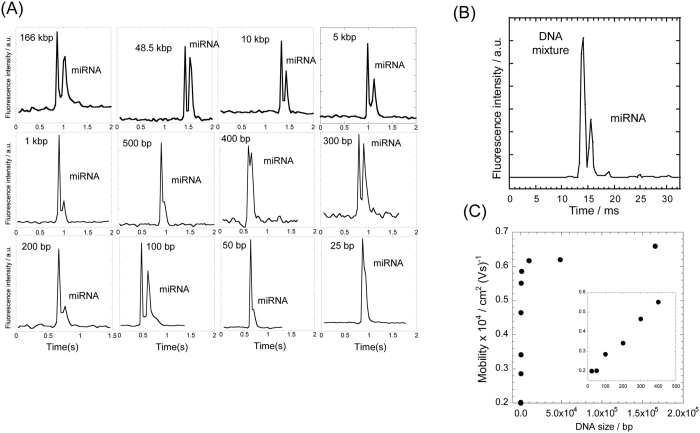
(**A**) Millisecond separation of miRNA, and various sizes of DNA, are shown in the electropherograms. Conditions: concentration of miRNA and DNA markers, 100 ng/μl and 20 ng/μl, respectively; separation field strength, 257 V/cm; working electrolyte, 5XTBE buffer (pH = 8.3); image capturing rate, 33 ms/frame. (**B**) Optimized millisecond separation of miRNA and mixture of DNA, ranging from 25 bp to 166 kbp, used in (**A**). The mixture was successfully separated within 20 ms with near baseline separation. Conditions: concentrations of miRNA and DNA mixture were 20 ng/μl and 2 ng/μl of each DNA marker used in Fig. 2(A), respectively; separation field strength, 533 V/cm; working electrolyte, 5XTBE buffer (pH = 8.3); image capturing rate, 250 μs/frame. (**C**) The electrophoretic mobilities of DNA markers in the hybrid structure region of nanopillars and nanoslits.

miRNA was spiked with the mixture of DNA markers ranging in size from 25 bp to 166 kbp, and separation experiments were performed using the 5XTBE buffer. To reliably identify DNA and miRNA, we used DNA stained with YOYO-1 and miRNA covalently bonded to Alexa Fluor^®^ 488. Figure 2A shows the separation results of miRNA (100 ng/μl) and a DNA marker (20 ng/μl) at 257 V/cm. These electropherograms were obtained using images captured at 100 μm from the end of the nanoslit region. Nearly complete separations were achieved within 1.5 s for as few as 100 bp. Each peak was assigned by varying the concentrations during the optimization process of the separation, indicating that DNA markers migrated faster than did the miRNA. Using two different staining dyes for DNA and miRNA may offer more reliable identification of the different types of nucleic acids. However, in this separation, dyes with nearly the same fluorescent wavelength of 520 nm were used for all the samples. This is because the two approaches commonly used for simultaneous two-color detection are an optical filtration method that involves changing observation filters, and recording through a 3CCD camera. However, neither of these methods could compare with the current capturing rate of sub-milli seconds and intrinsically reduce detectable photons. Therefore, peak assignments, based on the height and area of the peak, were adapted by changing the concentration of the input sample.

To separate the above DNA markers and miRNA in a single run, the electric field, concentration of the samples, image-capturing rate, sensitivity of the high-speed CCD camera, and gain of the image intensifier unit were comprehensively optimized to achieve separation at 20-ms. After optimizing separation conditions, a mixture of all the markers in a range from 25 bp to 166 kbp (2 ng/μL each), and the miRNA (20 ng/μL), were successfully separated within 20 ms at 533 V/cm, with a separation resolution of 1.67, as shown in Fig. 2B. To the best of our knowledge, this is the fastest complete separation of nucleic acids using microchip electrophoresis. In this separation, small peaks were observed at 18 and 25 ms. Because the value of the baseline noise was 1.3111, and peak intensities at 18 ms and 25 ms were 4.9 and 2.6, respectively, the peak at 25 ms may be noise (signal-to-noise ratio <3), and the peak at 18 ms may be a fragment of miRNA, which forms a different secondary conformation.

To evaluate the quality of miRNA separation, the resolution was calculated using the following equation:


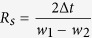


where *Δt* is the difference in retention time of sample 1 and 2, and *w*_*1*_ and *w*_*2*_ is the width of each sample peak, respectively. In the separation of DNA markers and miRNA mixtures, the resolutions of nearly all separations performed within 1.5 s were over 1.0 (Fig. 2A). The difference in mobility between the DNA markers and miRNA is a key factor that determines the resolution. The plotted mobilities of DNA markers, in the hybrid structure region of nanopillars and nanoslits, are shown in Fig. 2C; the dependence of mobility on the molecular weight of DNA was similar to that measured previously in free solution^20^,^21^. The electrophoretic mobility of DNA levels off and becomes constant when the DNA reaches molecular weights of ~170 bp^20^ or ~400 bp^21^ in the 1xTAE (40 mM) buffer. The absolute values of the electrophoretic mobility, calculated from 0.2 to 0.7 × 10^−4^ cm^2^V^−1^s^−1^, in our experiment, were approximately one tenth of those found in the literature, which ranged from 3.0 to 3.8 × 10^−4^ cm^2^V^−1^s^−1^. This difference may be attributable to the buffer constitute, 5xTBE, used in our study, while previous studies used 1xTAE. The capillary described in the literature was coated with neutral hydrophilic polymers such as polyacrylamide, but we used a high-concentration buffer to suppress the electroosmotic flow (EOF). This high-concentration buffer decreased the net charge of DNA molecules and slowed the velocity, decreasing the surface charge and suppressing the EOF.

### Separation of mixtures containing genomic DNA, total RNA, and miRNA

**Figure 3:**
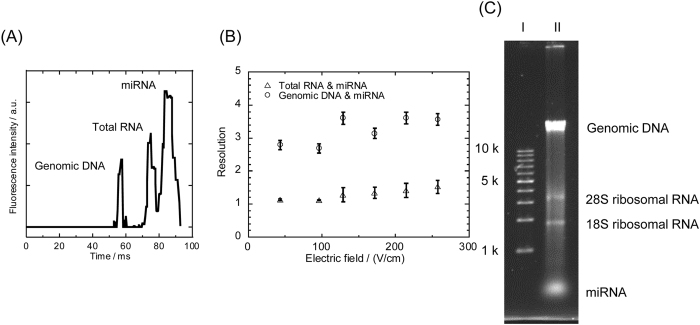
(**A**) Millisecond separation of a mixture consisting of miRNA, total RNA, and genomic DNA. Total RNA and genomic DNA were extracted from HeLa cells using a commercially available extraction kit. Conditions: concentrations of genomic DNA, total RNA, and miRNA were 20 ng/μl, 20 ng/μl, and 100 ng/μl, respectively; separation field strength, 533 V/cm; working electrolyte, 533 V/cm; image capturing rate, 500 μs/frame; dye, YOYO-1 for genomic DNA, SYBR Gold for total RNA, Alexa Flor^®^ 488 for miRNA (covalent bonding). (**B**) Resolution of separation of miRNA and genomic DNA (circle), and miRNA and total RNA (triangle). (**C**) An image of slab gel electrophoresis of molecular weight markers (1 kbp DNA ladder) in lane I and a mixture of genomic DNA and total RNA extracted from HeLa cells, and spiked miRNA, in lane II.

From a practical perspective, separating a mixture of genomic DNA, total RNA, and miRNA is more valuable for future development of on-chip diagnostics, such as extraction of miRNA from nucleic acid mixtures without conventional solid phase extraction and sequencing of a single miRNA on a single chip. Figure 3 shows separation of a mixture consisting of genomic DNA, total RNA, and miRNA from HeLa cells; 100 ms separation is demonstrated at 533 V/cm. The electrophoretic resolution of total RNA and miRNA, as well as HeLa genomic DNA and miRNA, was 2.14 and 4.30, respectively.

Through all of the experiments, relatively high concentration of miRNA, 100 μg, was required with that of samples of DNA and total RNA stained with YOYO-1 and STBR-Gold, respectively. This is because we used a fluorescence tag covalently bonded to miRNA, resulting in one fluorescent probe per miRNA and a weak fluorescent intensity.

### Separation mechanisms using a hybrid structure of nanopillars and nanoslits

Non-equilibrium transport in the nanopillars arranged in square array pattern, and entropic trapping in a nanoslit structure, were the two possible factors that presumably contribute to the millisecond separation of nucleic acids in the early stage of the experiments. We determined that non-equilibrium transport of DNA molecules in a square array pattern provides high-speed separation without losing the resolution of separation^22^. This theory of separation, proposed by Dorfman *et al*.^23^, states that under strong electric fields, larger polymers will elute faster from the nano-confinement, than will the smaller ones, via “torque-assisted escape”, if these polymers, such as DNA molecules, are sufficiently short to be rigid and straight. We have recently demonstrated that this theory can be applied to include molecules with weights of up to 1,000 bp; these do not behave as rigid and straight polymers, but the higher electric field (~500 V/cm) enhances the resolution of separation and shortens the time^24^. Because the hybrid structure of nanopillars and nanoslits converges with the electric field and generates a strong electric force, non-equilibrium transport was achieved in a few hundreds of a millisecond. Short and flexible miRNA may enhance the difference in mobility and lead to a successful high-speed separation; this is because single-stranded miRNA easily undergoes conformational fluctuation, even during electrophoresis, and easily entangles with the nanopillar. The molecular sieve effect may be used to retard the migration of miRNA.

When separating miRNA and larger DNA molecules sized over 1,000 bp, it is important to consider the mechanism of entropic trapping by the nanoslit structure. A separation technique, based on entropic trapping, was originally proposed by Han *et al*. in 2000; since then, other separation techniques have been derived from this method^25^,^26^. Large DNA molecules, with hydrodynamic gyration radii larger than the 100-nm nanoslit, are entropically unfavorable and tend to escape the nanoslit faster than does miRNA. However, in our hybrid structure, only one entropic barrier exists, and the nanoslit region is too long (~100 μm) to generate the difference in velocity, achieving the separation of miRNA and large DNA. Therefore, the contribution of entropic trapping may be smaller than that of other factors.

In addition to the non-equilibrium transport and the entropic barrier, the three-fold difference in the absolute mobility between miRNA and large-sized DNA, shown in Fig. 2, is an important substantial factor and may be directly linked to the speed of separation.

Interestingly, in this nanobiodevice, the resolution was lost around 400 to 500 bp, as shown in Fig. 2A. In theory, the total lengths of 400 and 500 bp are 136 and 170 nm, respectively; these lengths barely exceed the height of the nanoslit, which is 100 nm. It is possible that under conditions of physical confinement, large-sized DNA (400 and 500 bp) physically collides with the roof and floor of the nanoslit at a greater rate than does smaller-sized DNA (300 bp). Therefore, the mechanism of “torque-assisted escape” cannot be directly applied to DNA within this size range. This size criteria of “torque-assisted escape” agrees with our previous report^24^, which showed that the applicable size range can be expanded to nearly 1,000 bp inside a 4,000-nm-tall nanopillar array system. In this case, we can disregard the physical interaction of DNA in the direction of the z-axis. Therefore, entropic trapping and the difference in the absolute mobility, rather than torque-assisted escape, may be the dominant factor in achieving the separation.

Here, we developed a new device for sample injection; this approach achieves millisecond separation and eliminates the need for an offset microchannel in front of the region of separation. In microchip electrophoresis, it is important to manage band broadening of the injected sample through pinched or gated injection in order to achieve high resolution and speed of separation. A strong entropic barrier is present at the sample-loading side of the nanoslit region. Therefore, when the electric field was switched from sample-loading mode to separation mode, miRNA and DNA mixtures were transiently trapped at this side, and the concentration of the sample was increased as shown in Fig. 1C. This transient trap time is only submilliseconds-long because the strong electric field in the nanoslit region immediately attracts the samples; however, the narrow sample band contributes to the high resolution and speed of separation.

In conclusion, a novel hybrid structure of nanopillars and nanoslits, contained in a microchannel, can be applied as a matrix for the separation of nucleic acids. Using this approach, we achieved 20 ms and 100 ms isolation of miRNA from DNA fragments and nucleic acids, respectively. To achieve millisecond separation, it is important to position the hybrid structure in front of the separation channel with no offset from the sample-loading channel; this is where the sample is concentrated and where a high entropic barrier generates a narrow sample band near the hybrid structure. This rapid technique for miRNA separation does not employ chemical reagents and is important for miRNA sequencing using a semiconductor nanopore on a chip. Therefore, this technique may become key for integrating miRNA pretreatment process into a nanopore-based sequencer.

## Methods

### Reagents and materials

A mature sequence of let-7a (22 bases), covalently bonded with the fluorescence dye, Alexa Fluor^®^ 488 at the 5′ end (Integrated DNA technologies, Inc.), was used as a model of miRNA. Genomic DNA from HeLa, Jurkat (human acute T-cell leukemia), and NIH 3T3 (a mouse embryonic fibroblast line) cells was purchased from New England Bio Labs Japan Inc. and used as a model of genomic DNA. All tDNAs were isolated using a standard genomic purification protocol^27^, extracted with phenol, and equilibrated using 10 mM Tris-HCl (pH 7.5) and 1 mM EDTA. A mixture of total RNAs from a collection of adult human tissues, which serves as a standard for accurate and reproducible comparison of gene expression data in real-time quantitative PCR, was purchased from Clontech Laboratories, Inc.

Various sizes of DNA markers were used to assess the resolution of separation delivered by the nanobiodevice. T4 DNA (165.6 kbp) and λ DNA (48.5 kbp) were purchased from Nippon Gene Co., Ltd., Tokyo, Japan. DNA markers containing only a single size fragment (10 k, 5 k, 1 k, 500, 400, 300, 200, 100, 50, and 25 bp) were purchased from Thermo Fisher Scientific K.K, Tokyo, Japan. YOYO-1 (Life Technologies Japan Ltd., Tokyo, Japan) and SYBR Gold (Life Technologies Japan Ltd., Tokyo, Japan) were used for fluorescent labeling of the DNA and RNA markers, respectively.

### Fabrication of the device

The Cr layer (250 nm in thickness) was deposited onto quartz substrate (0.5 mm in thickness) using RF sputtering (SVC-700LRF, Sanyu Denshi); this layer was a mask for the dry etching process used to fabricate a microchannel. Positive photoresist (TSMR V50, Tokyo Ohka Kogyo Co.) was spin-coated onto the Cr layer at 500 rpm for 10 s and at 2000 rmp for 1 min. Then, the microchannel pattern, with a width of 25 μm, was formed using photolithography. After developing the resist (NMD-3, Tokyo Ohka Kogyo Co.), the patterned Cr area was etched by immersion in Cr etchant (H_2_O:Ce(NH_4_)_2_(NO_3_)_6_:HClO_4_, 85:10:5 by weight percent) for 5 min. The 2-μm-deep microchannel was formed using reactive ion etching (RIE-10NR, Samco Co.) at 150 W and CF4 flow rate of 15 sccm under ambient gas at 3.0 Pa; the etching time was 40 min. The inlet and outlet via holes (1.5 mm in diameter) for the microfluidic system were drilled with an ultrasonic driller (SOM-121, Shinoda Co.). After drilling the via holes, the residual Cr layer was lifted off with Cr etchant. A Cr layer (10 nm thick) was deposited within the microchannel. Positive resist (ZEP520 A7, Zeon Corp.) was coated on the microchannel by spin coating, and then the nanopillar pattern was drawn by electron beam lithography (SPG-724, Sanyu Electron Co.). After developing the resist (ZED-N500, Zeon Corp.), the Cr layer pattern was removed with Cr etchant. The 100-nm-deep microchannel was formed by using reactive ion etching at 150 W, with a CF_4_ flow rate of 15 sccm, under ambient gas at 3.0 Pa; the etching time was 2 min. After nanopillar etching, the residual Cr layer was lifted off with Cr etchant. The nanobiodevice was cleaned with piranha solution for 2 h and then bonded with a quartz cover glass by immersion in 55% H_2_SiF_6_ solution for 10 min and baking at 85 °C for 24 h in an oven under 20 MPa.

### Microchip electrophoresis

An inverted fluorescent microscope (Eclipse TE300, Nikon, Tokyo, Japan), equipped with a high voltage sequencer (HVS448-6000, Livermore, CA, Lab Smith), was used to perform high-speed electrophoretic separation. A concentrated buffer solution (5 × TBE; 445 mM Tris-Borate and 10 mM EDTA, pH 8.3, Sigma-Aldrich Co. LLC., Tokyo, Japan) was used to suppress strong electroosmotic flow in the quartz microchannel. A mercury lamp was used to observe the fluorescently stained DNA molecules and micro-RNA. To reduce photobleaching of the fluorescently stained DNA, total RNA, and miRNA, dithiothreitol (DTT, Sigma-Aldrich, Inc.) was added to the electrophoresis buffer at a final concentration of 10 mM. Unless specified otherwise, all microchip electrophoresis experiments used a 20 ng/μL solution of genomic DNA and DNA markers, 20 ng/μL solution of total RNA, and 10 ng/μL solution of miRNA.

Separation using the nanopillar and nanoslit structures was captured with a high-speed camera (HPV-2, Shimadzu) coupled with a high-speed gated image intensifier unit (modified for the high-speed camera, Hamamatsu Photonics K. K.), which can record videos at 1 million frames per second with a resolution of 312 × 260 pixels, using a 20×/0.45 NA objective lens (Nikon). After recording the video, fluorescent intensity, 100 μm downstream from the end of the nanostructures, was analyzed using image-processing software (Cosmos 32, Library, Tokyo, Japan), and electropherograms were obtained.

## Additional Information

**How to cite this article**: Wu, Q. *et al*. A millisecond micro-RNA separation technique by a hybrid structure of nanopillars and nanoslits. *Sci. Rep.* **7**, 43877; doi: 10.1038/srep43877 (2017).

**Publisher's note:** Springer Nature remains neutral with regard to jurisdictional claims in published maps and institutional affiliations.
